# Direct costs of acute respiratory infections in a pediatric long‐term care facility

**DOI:** 10.1111/irv.12350

**Published:** 2015-12-11

**Authors:** Meghan T. Murray, Elizabeth Heitkemper, Olivia Jackson, Natalie Neu, Patricia Stone, Bevin Cohen, Lisa Saiman, Gordon Hutcheon, Elaine L. Larson

**Affiliations:** ^1^ School of NursingColumbia University Medical CenterNew YorkNYUSA; ^2^ Elizabeth Seton Pediatric CenterYonkersNYUSA; ^3^ Department of PediatricsColumbia University Medical CenterNew YorkNYUSA; ^4^ Department of Infection Prevention and ControlNewYork‐Presbyterian HospitalNew YorkNYUSA

**Keywords:** Cost analysis, long‐term care, pediatrics

## Abstract

Acute respiratory tract infections (ARI) are a major burden in pediatric long‐term care. We analyzed the financial impact of ARI in 2012–2013. Costs associated with ARI during the respiratory viral season were ten times greater than during the non‐respiratory viral season, $31 224 and $3242 per 1000 patient‐days, respectively (*P* < 0·001). ARI are burdensome for pediatric long‐term care facilities not only because of the associated morbidity and mortality, but also due to the great financial costs of prevention.

## Introduction

Acute respiratory tract infections (ARI) account for an estimated 40% of all healthcare‐associated infections in pediatric long‐term care facilities (pLTCFs).^1^, ^2^ Residents in pLTCFs are at increased risk for ARI due to their complex and/or chronic medical conditions, frequent device utilization, immunologic immaturity, and behavioral factors.^2^, ^3^ Additionally, due to the home‐like environment, residents often receive care from a variety of providers including medical and nursing staff, therapeutic staff, school teachers and aides, and volunteers; these frequent interactions with multiple providers place the children at even greater risk for ARI.^2^ Outbreaks of ARI are common in pLTCFs and often affect a large proportion of the resident population, particularly during the respiratory viral season.^4^, ^5^ These infections are not only taxing on both the residents and the staff, but are likely a large financial and resource burden as well.^6^, ^7^

While previous studies have reported on the comorbidities, mortality, and hospitalizations associated with ARI, few, if any, studies have examined the financial burden of these infections in pLTCFs. The care of children with chronic and complex medical disorders is costly, but little is known about the added costs of ARI in this population.^8^ Therefore, the aim of this cost of illness study was to calculate the attributable cost of ARI in pLTCFs during the respiratory viral season as compared to the remainder of the year.

## Methods

### Study site and subjects

We conducted a retrospective cohort study from October 2012 to September 2013 at a 137‐bed pLTCF in the New York metropolitan area. The cost of illness study was conducted from the facilities perspective. Residents ranged in age from 2 months to 21 years (mean = 9·7 years) and length of stay ranged from 1 day to 21 years (mean = 4·4 years). Approximately 85% of residents had a gastrostomy, 51% had a tracheostomy, and 12% were on mechanical ventilation. Residents have the following comorbidities: 90% neurologic disorder (e.g., intraventricular hemorrhage, hypoxic ischemic encephalopathy, agenesis of corpus callosum), 64% pulmonary disorder (e.g., chronic lung disease, congenital respiratory failure, bronchopulmonary dysplasia), 25% cardiac disorder (e.g., congenital heart disease), 26% premature (i.e., <37 weeks gestational age), and 10% genetic disorders (e.g., trisomy 18, trisomy 21). All eligible residents receive seasonal influenza vaccination. This study was approved by our institutional review boards.

### ARI diagnostic testing

A respiratory illness algorithm is used to guide diagnostic testing at this facility. For residents who present with signs and/or symptoms of ARI for greater than 48 hours, direct fluorescent antigen (DFA) testing is performed. If DFA is negative and symptoms persist, a viral culture is performed. If the resident remains symptomatic and both the DFA and viral culture are negative, the reverse transcriptase polymerase chain reaction (RT‐PCR) testing is completed. All testing is performed at a referral laboratory.

### Data collection

The respiratory viral season was from October 2012 to May 2013, as determined by the Centers for Disease Prevention and Control.^9^ The non‐respiratory season was from June 2013 to September 2013. ARI were defined as clinician‐diagnosed respiratory tract infections identified from a line list maintained by the pLTCF's infection prevention and control coordinator. ARI included laboratory‐confirmed viral respiratory infections, source‐linked suspected viral respiratory infections, and respiratory infections with two or more signs or symptoms (i.e., cough, congestion, desaturations, diarrhea, fever, increased oxygen needs, increased secretions, vomiting, and/or wheezing) and negative diagnostic testing or no diagnostic testing sent. Confirmed and/or suspected bacterial infections were not included as an ARI (e.g., pneumonia, sinusitis, and tracheitis).

Attributable resources were collected for supplies related to infection prevention processes (i.e., personal protective equipment, hand hygiene, and environmental cleaning), ARI diagnostic testing (RT‐PCR, direct fluorescent antibody, and viral culture testing), antimicrobials prescribed for an ARI, influenza prophylaxis, and hours billed for consultation with the infectious disease physician retained by the facility. Medical charts for each resident with an ARI were reviewed for associated antimicrobial use and diagnostic testing. Residents' charts were also reviewed to determine whether they received oseltamivir, which was administered during outbreaks of influenza to all exposed residents, unless contraindicated.

Once all ARI resources were identified for the respiratory viral season and non‐respiratory viral seasons, the costs of each resource were estimated. To determine the associated costs of sanitation and environment cleaning, the quantity and cost of supplies associated with hand hygiene, contact and droplet isolation precautions, and terminal room cleaning were provided by the facility's purchasing department. These items included gloves, gowns, masks, hand sanitizer, and bleach wipes. The cost of diagnostic testing was provided by the laboratories contracted by the pLTCF. The vendors and item costs did not change during the study period. The costs of antibiotics and oseltamivir were calculated based on the lowest and most conservative estimate of the average wholesale price listed in the 2010 Red Book Drug Reference.^10^ Antibiotic and oseltamivir prices were standardized and inflation adjusted to 2013 using the Consumer Price Index (http://www.bls.gov/data/inflation_calculator.htm). A pediatric infectious disease consultant is employed by the pLTCF on an hourly basis to assist with the management of healthcare‐associated infections. This physician's hourly wages during the respiratory and non‐respiratory viral seasons were estimated from the Bureau of Labor Statistics Occupational Employment and Wages from May 2013 for pediatricians in New York State.^11^

### Data analysis

Descriptive analyses were conducted to summarize the differences in ARI costs across the respiratory and non‐respiratory viral seasons. Poisson regression was used to assess the difference in cost per resident‐day for influenza versus non‐influenza seasons. Statistical significance was set a priori to 0·05.

## Results

During the study period, there were 183 ARI in 127 residents: 137 during the viral respiratory season and 46 during the non‐respiratory viral season. Of the 183 ARI, 33 were due to a laboratory‐confirmed pathogen including 13 rhino/enterovirus, 9 human metapneumovirus, 3 parainfluenza, 3 coronavirus, 3 influenza, 1 RSV, and 1 adenovirus. Of the 3 confirmed cases of influenza, 2 were cases of influenza A and 1 was a case of influenza B. All cases of influenza occurred during the respiratory viral season. Sixty‐three infections had diagnostic testing that was negative and 87 infections were clinically diagnosed only. The costs associated with the respiratory viral season and the non‐respiratory viral season are summarized in the Table 1. The respiratory viral season was significantly more expensive than the non‐respiratory viral season (total costs: $31 224 and $3242 respectively, *P* < 0·001).

**Table 1:** Costs associated with acute respiratory tract infections (ARI) during respiratory and non‐respiratory viral seasons in a pediatric long‐term care facility, 2012‐2013

Categories	Respiratory viral season October 2012–May 2013	Non‐respiratory viral season June 2013 – September 2013
Personal protective equipment	$2245	$197
Hand hygiene	$128	$270
Environmental cleaning	$300	$232
Diagnostic testing^a^	$784	$505
Antimicrobials for ARI	$168	$33
Influenza prophylaxis	$27 338	$0
Infectious disease consultant	$262	$230
Total Cost	$31 224	$3242

Costs per 1000 resident‐days, USD.

a Includes reverse transcriptase polymerase chain reaction, direct fluorescent antibody, and viral culture testing.

## Discussion

In this study, the costs of the infection prevention and control measures expended were ten times greater during the respiratory viral season than during the non‐respiratory viral season. The vast majority of the cost burden, nearly 98%, was due to influenza prophylaxis. Our facility prophylactically treats all residents with oseltamivir at the onset of any confirmed case of influenza. During our study period, there were only three cases of influenza. However, this translated into all 137 residents receiving prophylaxis three times, thus resulting in high costs. The added costs of the other infection prevention and control activities we measured including diagnostic testing, physician consulting, and product usage, $644 per 1000 resident‐days, were similar to that reported in the pediatric acute care setting during RSV seasons ($760 per 1000 patient‐days).^6^ Surprisingly, the costs related to hand hygiene were greater during the non‐respiratory viral season as compared to the viral respiratory season. This may be due to an increase in gastrointestinal disease during the summer months (data not shown). While the cost effectiveness of oseltamivir has not been specifically evaluated in pLTCF, studies in the adult long‐term care setting have demonstrated that influenza prophylaxis results in cost savings to facilities overall.^12^

There are a few limitations to this study. Only direct costs were calculated, although the pLTCF suffers other losses not taken into account in this analysis. During outbreaks of ARI, for example, the facility's school is closed to prevent the transmission of infection. This results not only in a financial loss of state funding for days of closure, but also a psychosocial cost for the residents. Additional costs not accounted for include staff absenteeism and overtime, as well as psychosocial costs for the roommates of residents who have an ARI, as all residents within the affected room would be on contact/droplet isolation precautions. Costs of hospitalizations were also not included, as this study examined the costs for the facility only. Transfers to acute care would further increase the costs associated with ARI. For this analysis, the study period was limited to one year, which may not be fully representative of other years or sites.

ARI are burdensome for pLTCFs not only because of the associated morbidity and mortality, but also due to the great financial costs of prevention. Notably, the vast majority of the cost burden, $27 338 for facility‐wide influenza prophylaxis, can be avoided if no cases of influenza are introduced into the facility. Thus, further research should investigate ways to limit ARI transmission, specifically influenza, in this unique population and setting through measures such as improved hand hygiene and environmental cleaning, increased vaccination rates for facility staff and visitors, and use of masks during peak respiratory illness seasons.

## Funding

This work was supported by the Agency for Healthcare Research and Quality (R01 HS021470).
